# Eribulin targets a ch-TOG-dependent directed migration of cancer cells

**DOI:** 10.18632/oncotarget.6147

**Published:** 2015-10-19

**Authors:** Brice Chanez, Anthony Gonçalves, Ali Badache, Pascal Verdier-Pinard

**Affiliations:** ^1^ Centre de Recherche en Cancérologie de Marseille, Inserm, Marseille, France; ^2^ Institut Paoli-Calmettes, Marseille, France; ^3^ Aix-Marseille Université, Marseille, France; ^4^ CNRS, UMR7258, F-13009, Marseille, France

**Keywords:** eribulin, migration, microtubule, ch-TOG, EB1

## Abstract

Non-cytotoxic concentrations of microtubule targeting agents (MTAs) interfere with the dynamics of interphase microtubules and affect cell migration, which could impair tumor angiogenesis and metastasis. The underlying mechanisms however are still ill-defined. We previously established that directed cell migration is dependent on stabilization of microtubules at the cell leading edge, which is controlled by microtubule +end interacting proteins (+TIPs). In the present study, we found that eribulin, a recently approved MTA interacting with a new class of binding site on β-tubulin, decreased microtubule growth speed, impaired their cortical stabilization and prevented directed migration of cancer cells. These effects were reminiscent of those observed when +TIP expression or cortical localization was altered. Actually, eribulin induced a dose-dependent depletion of EB1, CLIP-170 and the tubulin polymerase ch-TOG from microtubule +ends. Interestingly, eribulin doses that disturbed ch-TOG localization without significant effect on EB1 and CLIP-170 comets, had an impact on microtubule dynamics and directed migration. Moreover, knockdown of ch-TOG led to a similar inhibition of microtubule growth speed, microtubule capture and chemotaxis. Our data suggest that eribulin binding to the tip of microtubules and subsequent loss of ch-TOG is a priming event leading to alterations in microtubule dynamics and cancer cell migration.

## INTRODUCTION

Natural products such as vinblastine and paclitaxel are important anti-tumoral drugs [1]. Their mode of action involves primarily the destruction of the microtubule cytoskeleton forming the mitotic spindle, leading to cell cycle arrest in mitosis and ultimately to cancer cell death. At usual clinical doses, common side effects of these chemotherapeutics are decreased neutrophil blood count, due to the targeting of the fast renewing hematopoietic tissues, and peripheral neurotoxicity which is thought to occur via the poisoning of microtubule-rich neuronal processes. Survival of cancer patients treated with microtubule targeting agents (MTAs) is frequently limited by tumor resistance to chemotherapies and progression towards invasive and metastatic grades. Detailed characterization of MTA binding to tubulin identified distinct binding pockets, offering alternative opportunities for treatments against tumor resistant to a site-specific class of MTAs [2]. Consequently, a continuous effort in natural product and medicinal chemistry generated a wealth of MTAs with a diverse array of chemical structures. Recently, eribulin (Halaven^®^), a synthetic analog of the sponge metabolite halichondrin B [3, 4], has been approved for the treatment of metastatic breast cancer patients previously treated with regimens including a taxane [5–7]. In phase III clinical trial, eribulin significantly improved overall survival of women heavily pretreated for metastatic breast cancer, highlighting a clinical benefit of eribulin after failure of other regimens.

Several arguments have been put upfront to challenge the idea that the antimitotic action is the sole mechanism by which MTAs impede tumor progression [8, 9]. Targeting the few rapidly dividing cells in human solid tumors cannot explain the effectiveness of these drugs, leading to the hypothesis that MTAs also target interphase cells [10]. This is supported by the observation that non-cytotoxic concentrations of MTAs inhibit intracellular trafficking [11]. Moreover, MTAs abrogate cancer and endothelial cell migration at doses that affect microtubule dynamics, without impacting microtubule mass [12]. Nevertheless, the underlying molecular mechanisms are still unclear, even though studies suggested that MTA might function by affecting localization of the EB1 +end-binding protein [13, 14]. The dynamics and stability of the microtubule cytoskeleton is mainly regulated by proteins interacting directly with microtubules and belonging to distinct functional categories [15]: nucleating proteins (including γ-tubulin), structural proteins (such as Tau and MAP2), motors (dyneins and kinesins) and +end tracking proteins or +TIPs (like EB1 or CLIP-170). We investigate how cues controlling the complex network of microtubule-associated proteins regulate cancer cell migration. In particular, we have characterized signaling pathways by which the ErbB2/HER2 receptor tyrosine kinase regulates the cortical localization of EB1-binding proteins, allowing capture and stabilization of EB1-decorated microtubules at the leading edge of migrating breast cancer cells [16–19]. We established that interference with +TIP expression or localization prevents chemotaxis of breast cancer cells in response to a gradient of heregulin (HRG), an ErbB receptor ligand.

The activity of eribulin on metastatic breast cancer may be due in part to an effect on cancer cell migration. In the present study, we have investigated the effect of eribulin on tumor cell motility and microtubule dynamics. We found that subnanomolar concentrations of eribulin impaired breast cancer cell chemotaxis and prevented microtubule extension towards the leading edge. The defect in peripheral microtubules was indistinguishable from the one generated by the disturbance of the membrane-associated protein complex and the +end complex that are involved in microtubule capture at the cell cortex [18]. Interestingly, eribulin treatment disturbed the +end localization of EB1 and CLIP170, but also of the tubulin polymerase ch-TOG. Finally, knocking down ch-TOG recapitulated the effects of anti-chemotactic concentration of eribulin suggesting that displacement of ch-TOG from microtubule tip might be a priming event in eribulin-induced disturbance of cancer cell migration.

## RESULTS

### Subnanomolar concentrations of eribulin altered cortical microtubules

**Figure 1:**
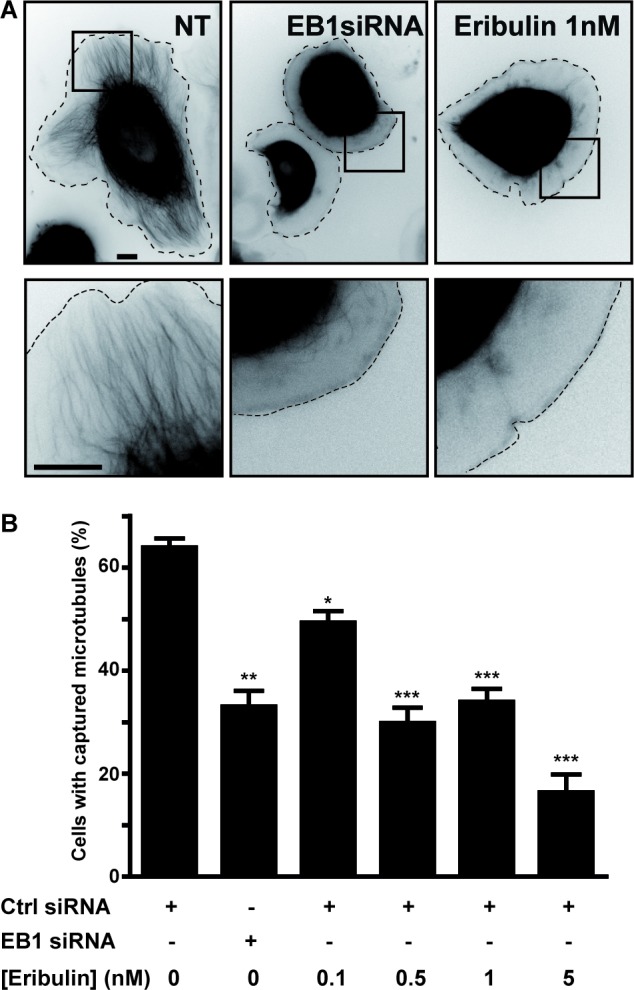
Eribulin prevents microtubule capture at the cortex of SKBr3 cells SKBr3 cells, transfected with EGFP-tubulin and a control (Ctrl) or an EB1 siRNA for 48 h, were grown in the presence of DMSO 0.5% (NT; not treated with eribulin) or eribulin for 4 h, before addition of HRG to trigger formation of membrane protrusions. **A.** Representative snap-shots of SKBr3 cells (upper panels) with captured (NT) or non-captured (EB1 siRNA; 1 nM eribulin) microtubules. Zooms on the peripheral regions indicated by the black squares are presented in the lower panels. Black scale bars represent 10 μm. Dashed lines indicate cell limits. **B.** The percentage of cells with captured microtubules was determined in three independent experiments (from a total of 150 cells) and presented as mean +/− SD; Student t-test with Welch correction: *p < 0.05, **p < 0.01, ***p < 0.001.

We performed a cytotoxicity assay on three different breast cancer cell lines representative of various molecular breast cancer subtypes, and compared the effects of eribulin with those of other MTAs, i.e. paclitaxel and vinblastine (Figure S1). The ErbB2-overexpressing SKBr3 cells were the most sensitive to eribulin, relative to the EGFP-tubulin and estrogen-receptor positive T47D cells and triple-negative MDA-MB-231 cells (Figure S1A; IC_50_ of 0.3, 1.0, 1.5 nM for SKBr3, T47D and MDA-MB-231 cells, respectively) and eribulin was more cytotoxic (IC_50_ 0.3 nM) than vinblastine (IC_50_ 0.9 nM) and paclitaxel (IC_50_ 2.2 nM) in SKBr3 cells (Figure S1B). Since the SKBr3 cells are a model cell line for investigating the role of microtubules in cell migration, we focused our study on this cell line. Upon addition of HRG, an ErbB2-activating ligand, SKBr3 cells form wide protrusions, populated by microtubules that extend to the cell periphery [16, 17]. Treatment with subnanomolar concentrations of eribulin for 4 hours was not cytotoxic (Figure S2) and did not affect HRG-induced membrane protrusions, but strongly inhibited the extension of microtubules in these protrusions (Figure 1A). This was reminiscent of the phenotype observed when the interaction between the MT-capture complex at the plasma membrane and the +TIP complex was disturbed [18]. Therefore, we evaluated the number of cells displaying a captured microtubule phenotype (i.e. cells with a large number of microtubules extending within protrusions) vs. the number of cells in which microtubules remained at a distance from the cell periphery. The percentage of cells displaying microtubule capture decreased in a dose-dependent manner starting from eribulin doses as low as 0.1 nM (Figure 1B). Noticeably, at 0.5 nM, eribulin reduced the percentage of cells with captured microtubules as efficiently as the knockdown of EB1, a master +TIP (Figure 1B).

### Eribulin prevented chemotaxis of breast cancer cells

**Figure 2:**
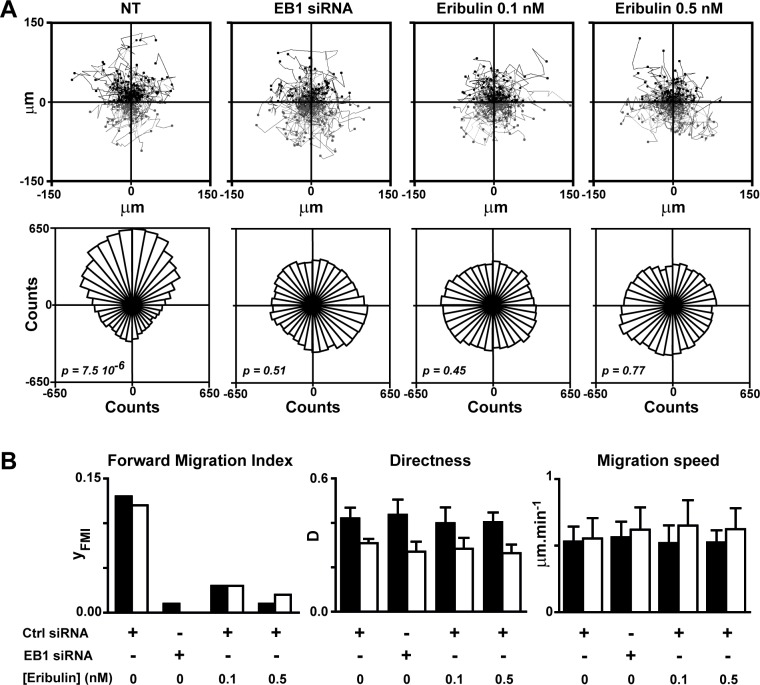
Eribulin inhibits HRG-induced chemotaxis SKBr3 cells were transfected with a control (Ctrl) or an EB1 siRNA for 48 h before tracking of cells in Dunn chambers for 4 or 8 h, as they migrated in response to a HRG-gradient (highest concentrations at the top of the figure). **A.** Upper panel: tracks of individual cells set to the same origin; cells migrated towards high and low HRG concentrations are represented in black and grey, respectively. Lower panel: rose plots reflecting cell distribution after 4 h of migration; p value < 0.05 indicates unimodal directional cell distribution in the Rayleigh test. NT, not treated with eribulin. **B.** Cell forward migration index (y_FMI_), cell directness (D) and migration speed were calculated after 4 h (black bars) or 8 h (white bars) of migration. Mean and SD from three independent experiments and at least 150 cells are presented. See Supplemental Material and Methods for definitions and equations for each parameter.

We previously established that defects in microtubule capture at the cell cortex led to strongly disturbed HRG-induced chemotaxis of breast cancer cells. Thus, we measured the impact of eribulin on chemotaxis, by tracking SKBr3 cells as they migrated in response to an HRG gradient for 4 hours (Figure 2A) and 8 hours (Figure S4). We determined that exposure to 0.1 nM eribulin was sufficient to strongly limit the efficiency of cell forward migration (Figure 2B) and completely disorient migrating cells (Figure 2A), similarly to EB1 down-regulation. Interestingly, eribulin did not affect other parameters of cell motility such as cell migration speed and directness, even when increasing the concentration of eribulin to 0.5 nM and the exposure time to 8 hours (Figure 2B).

### Inhibition of chemotaxis by eribulin occurred in the absence of detectable effects on EB1 and CLIP-170 comets

**Figure 3:**
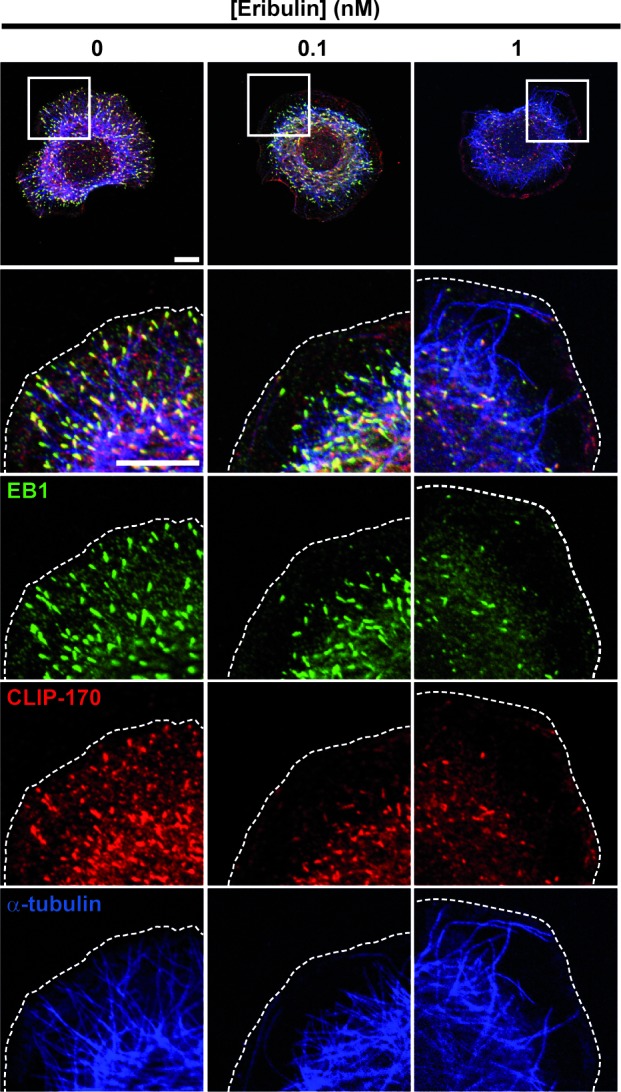
Effects of eribulin on EB1 and CLIP-170 microtubule +end localization Following treatment with eribulin for 4 h, SKBr3 cells were stimulated with HRG for 30 min and fixed. EB1, CLIP-170 and tubulin localizations were visualized by triple immunofluorescence labeling. White scale bars represent 10 μm; cell limits are delineated with dashed lines.

**Figure 4:**
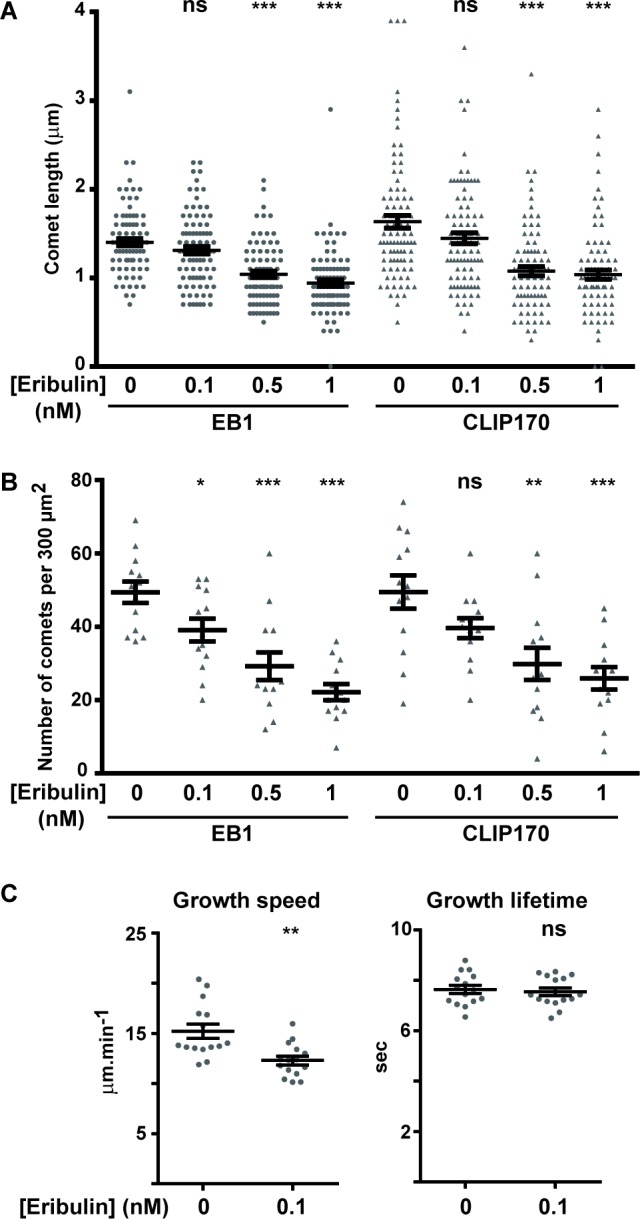
Effects of eribulin on the length and density of EB1 and CLIP-170 comets, and on microtubule dynamic parameters The length and density of fluorescent EB1 and corresponding CLIP-170 comets were measured on immunofluorescence images such as depicted in Figure 3. **A.** For each condition, a total of 90 comets from three independent experiments (10 comets per cell, 3 cells per experiment) were measured. **B.** For each condition, a total of 13 cells from five independent experiments were analyzed for the number of EB1 or CLIP-170 comets in an area of 300 μm^2^. **C.** EB1-GFP comets were tracked in HRG-stimulated SKBr3 cells, pre-treated or not with 0.1 nM eribulin for 4 h, and resulting tracks were analyzed using +TipTracker. Growth speed (including pauses) and growth lifetime were calculated. For each condition, a total of >14,000 tracks, in 15 cells from 3 independent experiments were analyzed. Medians and SD are presented, Student t-test with Welch correction: ns > 0.05, *p < 0.05, **p < 0.01, ***p < 0.001.

Since exposure to subnanomolar concentrations of eribulin and EB1 knockdown have similar effects on cortical microtubules and directed cell migration, we analyzed whether eribulin treatment affected the function of EB1 and of the microtubule- and EB1-binding protein CLIP-170. Eribulin did not alter the expression levels of EB1 or CLIP-170 (Figure S3B). Localization of EB1 and CLIP-170 by immunofluorescence revealed an evident loss of these proteins from microtubule +ends when cells were treated with 1 nM of eribulin (Figure 3). At 0.1 nM of eribulin, despite an obvious defect in microtubule capture at the cortex, microtubules still appeared decorated with EB1 and CLIP-170 comets. To quantify the impact of subnanomolar concentrations of eribulin on +TIP comets, we measured the length (Figure 4A) and the number (Figure 4B) of EB1 and corresponding CLIP-170 comets. The density of EB1 comets was strongly decreased starting from 0.5 nM eribulin; EB1 comet number was slightly decreased at 0.1 nM of eribulin, but this was not significant for the corresponding CLIP-170 comets (Figure 4B). The length of the remaining EB1 and CLIP-170 comets was also considerably reduced above 0.5 nM eribulin; but it was not significantly impacted by 0.1 nM of eribulin (Figure 4A). Collectively, our results show that the inhibition of microtubule capture and chemotaxis by 0.1 nM of eribulin is not associated with a detectable change in the length and density of +TIP comets, and thus cannot be merely explained by the loss of EB1 or EB1-associated +TIPs.

### Anti-chemotactic eribulin dose reduced microtubule growth speed

Since 0.1 nM eribulin treatment disturbed microtubule capture, we examined whether this might be a consequence of altered microtubule cytoskeleton dynamics in SKBr3 cells. Because the treatment did not change EB1 comets, we measured microtubule dynamics by tracking EB1 comets in SKBr3 cells stably expressing EB1-GFP. We have previously verified that the SKBr3.EB1-GFP cell line had the same sensitivity to eribulin than parental SKBr3 cells (Figure S1C, IC_50_ value of 0.3 nM). Using the +TipTracker software, we calculated multiple parameters of microtubule dynamics and in the presence of eribulin at 0.1 nM, we observed an increase in the percentage of slow microtubule tracks (Figure S5) and globally a significant decrease in microtubule growth speed (Figure 4C) and growth length (data not shown) relative to control, with no significant change in the growth lifetime (Figure 4C) or in rescue and catastrophe frequencies (data not shown).

### Recruitment of ch-TOG at microtubule +ends was altered by the anti-chemotactic dose of eribulin

**Figure 5:**
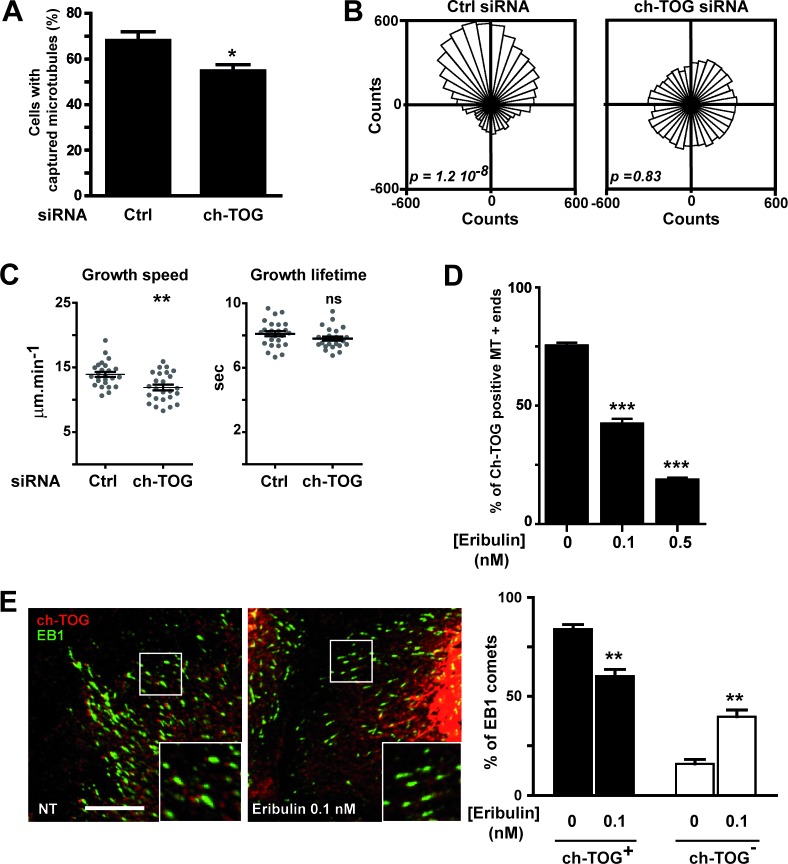
Eribulin disturbs the microtubule +end localization of ch-TOG, which is involved in MT dynamics and chemotaxis **A.**-**C.** SKBr3 cells were transfected with a control (Ctrl) or a ch-TOG siRNA before analysis of **(A)** microtubule capture at the cell cortex, **B.** chemotaxis and **C.** microtubule dynamics as described in Figure 1B, 2A and 4C, respectively. **D.**, **E.** 0.1 nM eribulin affects microtubule +end localization of ch-TOG. **D.** The presence of ch-TOG labeling at the tip of microtubule ends was assessed, in the presence or absence of eribulin, on a total of 50 microtubules in the periphery of SKBr3 cells displaying double fluorescent labeling for tubulin and ch-TOG. The percentage of ch-TOG positive microtubules was determined in two independent experiments with ten cells per condition in each experiment. **E.** chTOG tip labeling and EB1 comets were identified by dual fluorescent labeling (left panel). Exposure time was the same for all conditions analyzed for each fluorescence channel. In contrast to control cells (NT; non treated with eribulin), in cells treated with eribulin, many EB1 comets did not show the typical ch-TOG tip labeling, as observed in zoomed areas (insets). White scale bar represents 10 μm. The percentage of EB1 comets also displaying ch-TOG labeling was determined in a 900 μm^2^ area in five cells per condition (right panel). Student t-test with Welch correction: ns > 0.05, *p < 0.05, **p < 0.01, ***p < 0.001.

The effect of 0.1 nM eribulin treatment on microtubule capture and directed migration did not correlate with the loss of EB1 or EB1-associated proteins. Thus, we searched for other possible eribulin effectors that could mediate its effects on microtubule capture. Among +TIPs, ch-TOG was a relevant candidate: it is a tubulin polymerase associating with the very tip of microtubules, which tracks microtubule +ends independently of EB1 [20–22]. We observed that in SKBr3 cells ch-TOG had a specific spot-like localization clearly distinct from EB1 comet-like labeling (Figure S6 and 5E). We found that when ch-TOG expression was down-regulated (Figure S3A), microtubule capture at the cell cortex was decreased by 20% (Figure 5A) which was equivalent to the effect of 0.1 nM eribulin-treatment (Figure 1B). Down-regulation of ch-TOG also prevented HRG-dependent chemotaxis (Figure 5B), with no effect on cell migration speed (data not shown). Noticeably, down-regulation of ch-TOG significantly reduced microtubule growth speed (Figure 5C). These similarities between the effects of 0.1 nM eribulin treatment and depletion of ch-TOG on microtubule dynamics and cancer cell migration raised the possibility that eribulin disturbed ch-TOG localization or function. Thus, we analyzed the impact of eribulin on ch-TOG localization at the tip of microtubules (Figure 5D and 5E). We observed a strong reduction of the number of ch-TOG positive microtubule +ends, with a 44% decrease at 0.1 nM of eribulin compared to untreated cells (Figure 5D). We have verified that eribulin treatment was not altering the expression of ch-TOG (Figure S3C).

We further assessed that 0.1 nM-eribulin was primarily delocalizing ch-TOG rather than EB1 from microtubule +ends by determining the number of EB1 comets harboring a ch-TOG labeling in treated and untreated cells (Figure 5E). We observed that in treated cells, many microtubule +ends had lost ch-TOG labeling, but still harbored EB1 comets: there was 25 % less ch-TOG labeled EB1 comets in eribulin treated cells relative to untreated cells. These results indicate that low doses of eribulin disturbed the recruitment of ch-TOG to microtubule +ends and that the loss of ch-TOG induced a decrease in microtubule growth speed and in microtubule capture, and abrogated directed migration.

## DISCUSSION

The present study shows that in non-cytotoxic conditions, eribulin altered breast cancer cell migration by specifically inhibiting chemotaxis towards HRG. This effect was correlated with reduced microtubule growth rate and defective microtubule capture at the cell cortex. As MTAs were previously shown to alter EB1 localization, we explored the impact of eribulin on EB1. The anti-chemotactic dose of 0.1 nM eribulin had no significant effect on EB1 and CLIP-170 localization, but induced a detectable loss of ch-TOG from the microtubule +ends. In addition, the down regulation of ch-TOG also led to inhibition of microtubule growth rate, microtubule capture and chemotaxis. Collectively, these results suggest that eribulin-dependent delocalization of ch-TOG from microtubule +end is sufficient to decrease microtubules growth speed, independently of EB1, which in turn affects microtubule capture at the cell cortex and specifically prevents steering of cells in the direction of the chemoattractant.

The observation that low doses of eribulin affect primarily ch-TOG is consistent with recent models suggesting that ch-TOG and EB1 bind to separate loci on microtubule ends: ch-TOG associates with the frayed tip of microtubules, whereas EB1 requires complete lateral interactions of protofilaments [20, 23]. Yet the two proteins are linked functionally, as the active incorporation of GTP-tubulin dimers by ch-TOG maintains the generation of new binding sites for EB1. This is probably why we observed a strong decreased in the number of EB1 comets upon ch-TOG knockdown (Figure S6) and upon treatment with high doses of eribulin that have a drastic effect on ch-TOG localization (Figure 5D).

Eribulin appears to impact microtubule dynamics slightly differently than many antimitotic drugs, such as vinblastine, by not altering catastrophe frequencies [4, 24]. In addition, eribulin inhibits the binding of vinblastine to tubulin non-competitively indicating a different site of interaction on β-tubulin [25]. *In vitro*, eribulin acts by specifically affecting growth phases, in accordance with our results in cells where it decreased microtubule growth rate (Figure 4C) and length (data not shown). Eribulin does not affect dynamic instability at microtubule minus ends. It was also calculated that it binds to microtubule with a maximum of 15 sites per microtubule [24]. These pharmacological characteristics suggest that eribulin targets more specifically the microtubule +ends.

**Figure 6:**
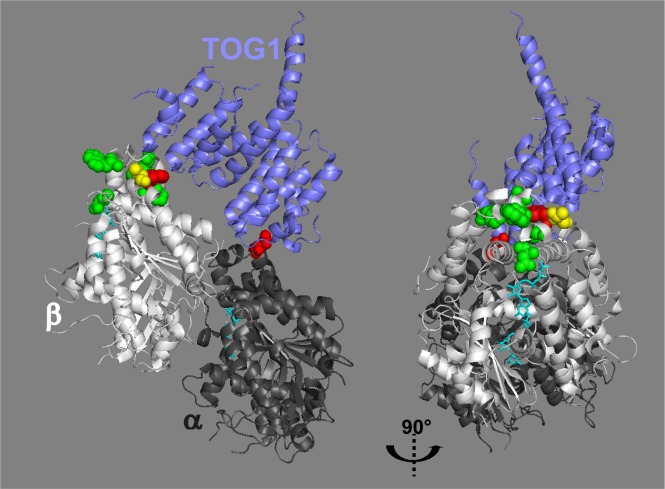
The predicted eribulin binding site and the TOG-β-tubulin interface are juxtaposed The model of the TOG1-tubulin complex depicted by Ayaz et al [27] (PDB 4FFB) was used to highlight the amino-acid residues (sphere representation) of tubulin that were predicted to be in contact with eribulin [26] in green, in contact with TOG1 in red or in contact with both in yellow. In the right panel, the model has been rotated 90° to the right. GTP molecules are shown in stick representation (cyan).

We observed that ch-TOG localization at the microtubule ends is remarkably sensitive to eribulin treatment, relative to other +TIPs. How does eribulin induce the loss of ch-TOG from microtubule +ends? We speculate that by poisoning microtubule +ends, eribulin binding to tubulin at substoichiometric concentrations [4, 24] disturbs the binding of the tubulin polymerase ch-TOG, thereby impacting tubulin polymerization which translates in a decrease of microtubule growth speed; this does not require the delocalization of EB1 which occurs at higher eribulin concentrations in our cancer cell model. Molecular docking suggests that halichondrin B/eribulin binds near β-tubulin loops H11-H11′ and H3′-H3 at the interdimer interface [26] (Figure S7). Highlighting the amino-acid residues of β-tubulin that are predicted to be in contact with eribulin [26] and the amino-acid residues of β-tubulin involved in the interaction with TOG1 domain [27], shows that the presumed binding pocket of eribulin and the β-tubulin-TOG interface are in close vicinity (Figure 6). Therefore we speculate that, upon binding to β-tubulin, eribulin induces a conformational change that destabilizes the ch-TOG-tubulin complex. Recently, co-crystallization of tubulin dimers as parts of a TTL-T2S complex with rhizoxin, maytansine or PM060184 revealed a previously undescribed binding site on β-tubulin [28]. This site is shaped by loops S3-H3, S5-H5 and H11-H11′ and is located nearby the eribulin/halichondrin B docking site (Figure S7), suggesting that eribulin belongs to the same family of MTAs. Binding of MTAs at this site would directly prevent the addition of tubulin dimers at the +end. Noticeably, T-DM1, a maytansinoid conjugated to Trastuzumab was approved specifically for the treatment of ErbB2/HER2-positive metastatic cancer [29], indicating the potential benefit of targeting this novel MTA binding site. Further experimentation will be necessary to investigate how eribulin affects the interaction of ch-TOG with the +end of microtubules and/or with tubulin dimers.

We previously found that localization at the cell cortex of the EB1-interacting proteins APC (adenomatous polyposis coli) and the spectraplakin ACF7 is required for both microtubule capture and chemotaxis [18, 19], underpinning a central role of EB1-mediated microtubule anchoring at the leading edge for directed migration. How does the loss of ch-TOG from microtubule +ends lead to a defect in microtubule capture? Ch-TOG might mediate the interaction of microtubules with proteins localized at the cell cortex and thereby participates to microtubule capture and stabilization. While we cannot exclude that ch-TOG associates directly with cortical components, microtubule +end cortical interactions are more likely driven by EB1, which is known to bind SxIP motif-containing cortical proteins [18, 22, 30–32]. Reconstitution experiments strongly suggest that ch-TOG allosterically enhances the binding of EB1 to the microtubule +end [22]. This is consistent with the loss of EB1 from microtubule +ends upon down regulation of ch-TOG in primary neurons, NIH-3T3 fibroblasts [33, 34] and our own breast cancer cell model (Figure S6). Thus, the impact of eribulin on microtubule capture might be the consequence of indirectly disturbing EB1 localization, as a result of the loss of ch-TOG. These results also suggest reconsidering the mechanism whereby low concentrations of other MTAs, that decrease EB1 comet length, affect endothelial and cancer cell migration [13, 14, 35].

Because SLAIN1 and 2 proteins were shown to act as crosslinkers between EB1 and ch-TOG and participate to the recruitment of ch-TOG to microtubule +ends [33], one cannot exclude their possible contribution to eribulin's effect. However, it was observed that, in HeLa cells where endogenous SLAIN proteins are expressed at low levels, ch-TOG was not recruited to EB1 comets, but was distinctively detected ahead of EB1 comets; strikingly, overexpression of SLAIN led to perfect comet-like co-localization of ch-TOG and EB1 [20]. The localization of ch-TOG ahead of EB1-comets in SKBr3 cells suggests that SLAIN proteins do not have a major impact on ch-TOG recruitment on microtubule +ends. In addition, downregulation of both SLAIN1 and 2 in neurons did not affect microtubule growth rate; whereas downregulation of ch-TOG induced a decrease in microtubule growth rate in neurons [32] and in many other cellular models [32, 33, 35], including ours. Altogether these data indicate that eribulin primarily displaces ch-TOG from microtubule +ends.

While EB1 might mediate some of the effects of eribulin on microtubule capture and chemotaxis, it is important to observe that at 0.1 nM, eribulin barely affected EB1 comet density and length, but prevented directed migration, suggesting an alternative mechanism. Interestingly, this low dose of eribulin induced a decrease in microtubule growth speed, similar to the one observed upon ch-TOG down regulation. This raised the possibility that the change in microtubule growth speed was responsible for preventing microtubule capture at the cell cortex. We propose that directed migration rely on a tight synchronization of microtubule assembly and leading edge progression. Thus, slowing down microtubule growth at protrusion inception would disconnect microtubules from the fast forward progression of the leading edge, thereby preventing their capture. In vitro, ch-TOG strongly synergizes with EB1 for catalyzing the tubulin incorporation at microtubule +ends to reach the 10-20 μm.min-1 microtubule growth speeds observed in cells [22], indicating that it is a key physiological regulator of microtubule growth. A recent study highlighted the importance of a tight control of microtubule growth speed during mitosis [36]. Indeed, colorectal cancer cell lines presenting chromosome instability showed high microtubule growth speed that was dependent on the activity of the TACC3-ch-TOG complex. Interestingly, microtubule growth speed could be brought back to values found in chromosomally stable cell lines by either down-regulation of ch-TOG or by subnanomolar concentrations of microtubule interacting agents. Therefore, microtubule growth speed may be a key factor for the regulation of microtubule capture at both chromosome kinetochores during cell division and cell cortex during cancer cell migration.

The present study highlights that impairing specifically the dynamics of the microtubule +end by disturbing the associated protein, ch-TOG, inhibits some aspects of cancer cell migration. Thus, further discovery and development of compounds targeting specifically microtubule +end, and the interaction of ch-TOG with tubulin, may provide treatments of advanced cancers therapeutics with improved therapeutic windows.

## MATERIALS AND METHODS

### Cell culture

SKBr3, MDA-MB-231 and T47D breast cancer cell lines, purchased from ATCC-LGC standards (Molsheim, France), were cultured in DMEM media supplemented with 10% FBS, sodium pyruvate at 37°C and in a humidified atmosphere with 5% CO_2_.

### Cytotoxicity assay

Eribulin mesylate (Eisai, Research Triangle Park, NC), paclitaxel and vinblastine (Sigma-Aldrich, St Louis, MO) were dissolved in DMSO and stored as a 5 mM stock in glass vials at −20°C. Serial dilutions of drugs were performed in 50% DMSO in glass vials and stored as 100-fold stock at −20°C. Wells of 96-well plates were coated with 25 μg/ml rat tail collagen I (Roche Diagnostics, Mannheim, Germany) in PBS, prior to seeding with 5000 cells per well. Effect of the drugs on cell viability was assessed with a sulforhodamine B assay [37] after 72 h of drug exposure. The final concentration of DMSO (0.5%) was the same for both drug-treated and control cells.

### Immunocytochemistry

SKBr3 cells were seeded at 10^5^ cells per well in a 12-well plate containing collagen-coated glass coverslips. After 48 h of culture, cells were treated with drugs (or DMSO) for 4 h before addition of 1 nM of heregulin β1 (HRG; R&D Systems, Abingdon, United Kingdom) with DMSO or drug for 30 min. Cells were fixed with methanol containing 1 mM EGTA at −20°C for 10 min, followed by 4% formaldehyde in PBS for 20 min at room temperature. In the case of ch-TOG labeling, cells were simply fixed with methanol at −80°C for 10 min. Immunolabeling was performed with antibodies against EB1 (Santa Cruz Biotechnology, Dallas, TX), CLIP-170 (Synaptic Systems, Goettingen, Germany), α-tubulin (Sigma-Aldrich) or ch-TOG (a generous gift from Dr Lynn Cassimeris, Lehigh University Bethlehem, PA) and secondary antibodies labeled with DyeLight^®^ 405, AlexaFluor^®^ 488 or 594 (Jackson ImmunoResearch Europe, Suffolk, UK). Images were acquired on a structured light ApoTome™ microscope (Zeiss, Münich, Germany) equipped with a 63x 1.4 plan ApoChromat objective and an Axiocam™ MRc5 camera.

### Western blotting

Cells were lysed in a NP-40 based lysis buffer (50 mM Tris pH 7.5, NP-40 1%, 120 mM NaCl, 0.5 mM EDTA, 1 mM DTT) supplemented with protease and phosphatase inhibitors (Roche Diagnostics). Samples were prepared as described previously [19]. Samples were run on Novex NuPAGE^®^ Bis-Tris 4-12% polyacrylamide gels (Life technologies, Carlsbad, CA) using a MOPS based running buffer (50 mM MOPS, 50 mM Trisbase, 0.1% SDS, 1 mM EDTA, pH 7.7). Proteins were transferred onto nitrocellulose membranes and detected by chemoluminescence using primary antibodies against EB1 (Pharmingen, BD Biosciences, San Diego, CA), α-tubulin (Sigma-Aldrich), and ch-TOG (Ecrins Therapeutics, Grenoble, France) and secondary antibodies coupled to HRP.

### Transfection of siRNA and cDNA

Transfection of siRNA (30 pmoles) and cDNA (3 μg) was performed by nucleofection (kit V) as recommended (Lonza, Cologne, Germany). siRNA against EB1 (sense strand: *UUAAAUACUCUUAAGGCAUTT*), ch-TOG (sense strand: *GAAAUACUCUUAAUUCUAATT)* and LacZ (control siRNA; sense strand *GCGGCUGCCGGAAUUUACCTT*) were synthesized by Life technologies. Efficiency and specificity of siRNA are presented in figure S3A. cDNA coding for EGFP-α-tubulin was obtained from Clontech (Mountain View, CA); mCherry-α-tubulin was a gift from Gia Voeltz, University of Colorado, Boulder (Addgene plasmid # 49149).

### Quantitation of the cell population with captured microtubules by time-lapse video microscopy

SKBr3 cells expressing EGFP-α-tubulin were either transfected with the indicated siRNA for 48 h, or pretreated with 0.5% DMSO alone or with eribulin for 4 h, before stimulation by 5 nM HRG and determination of the percentage of cells showing captured microtubules over a period of observation of 30 min. Detailed procedure has been described previously [17].

### Migration in Dunn chambers, cell tracking and quantitation of chemotaxis

SKBr3 cells plated on collagen-coated coverslips were exposed to a HRG gradient (10 nM HRG in the outside well) in Dunn chambers. SKBr3 cells were tracked for 4 to 8 h in the presence of eribulin. Detailed procedure has been described previously [19]. Tracks were analyzed using the Chemotaxis and Migration Tool of ImageJ to calculate cell migration speed, directness and forward migration index along the axis parallel to the gradient (y_FMI_). The Rayleigh test for unimodal clustering of directions was used to test directionality in the HRG gradient. Distribution of directions was considered uniform (random migration) for p>0.05. Definitions and equations used to calculate these migration parameters are presented in Supplemental Material and Methods.

### Measure of microtubule dynamic parameters

SKBr3 cells stably expressing EB1-GFP (SKBr3.EB1-GFP) were grown on collagen-coated glass bottom dishes (Greiner Bio-One, Frickenhausen, Germany) for 48 h. Medium was changed for DMEM^gfp^-2 (Evrogen, Moscow, Russia) supplemented with 5 nM HRG. After 10 min, images of EB1-GFP comets were acquired every 60 ms during 1 min, with a confocal spinning-disk microscope (Zeiss) equipped with a 100x-oil objective and a 491 nm laser with power tuned at 30%. For assessing microtubule dynamics, images were analyzed with the MatLab plug-in plusTipTracker [38]. The quality of the movies was assessed by examining comet detection and track linkage performance. Movies with high rate of false positive or false negative detection were not used for tracking. The same parameters were used for all movies: search radius range: 5 to 12 pixels; minimum track length: 4 frames; maximum gap length: 12 frames; maximum forward angle, 50°; maximum backward angle, 10°; maximum shrinkage factor: 1.5; fluctuation radius, 1.5 pixels. Frame rate: 0.8 s^−1^ and pixel scale: 133 nanometers.

### Quantitation of +TIP localization on microtubule +ends

EB1 and CLIP-170 comet length was measured using ImageJ and a line histogram plug-in (George Patterson, Biophotonics Section, NIH). Each comet was measured from head to tail using pixels with intensities above 15. EB1 and CLIP-170 comet density in SKBr3 was estimated using the spot detector function of freeware ICY (icy.bioimageanalysis.org). Background noise was reduced with ImageJ and a Gaussian filter was applied over images with ICY. Spot detection was calibrated with size filtering: from 3 to 3000 pixels; and scale: 3-7 pixels. The EB1 and CLIP 170 comets detection was done over an identical 300 μm^2^ region of interest in protrusions. Images were checked for false positive and false negative detection. The presence of ch-TOG labeling at the tip of microtubule +ends at the periphery of SKBr3 cells was manually assessed on images displaying double fluorescent labelling for tubulin and ch-TOG. The percentage of ch-TOG positive microtubules was determined on a total of 50 microtubules per cell in ten cells per condition in two independent experiments. In an independent experiment, the percentage of EB1 comets displaying a ch-TOG labeling, irrespective of its fluorescence intensity, was determined in a 900 μm^2^ area in five cells per condition. In individual experiments, exposure time for each fluorescence channel was the same for each condition analyzed.

## SUPPLEMENTAL MATERIALS METHODS AND FIGURES



## References

[1] Mukhtar E, Adhami VM, Mukhtar H (2014). Targeting microtubules by natural agents for cancer therapy. Mol Cancer Ther.

[2] Perez EA (2009). Microtubule inhibitors: Differentiating tubulin-inhibiting agents based on mechanisms of action, clinical activity, and resistance. Mol Cancer Ther.

[3] Towle MJ, Salvato KA, Budrow J, Wels BF, Kuznetsov G, Aalfs KK, Welsh S, Zheng W, Seletsky BM, Palme MH, Habgood GJ, Singer LA, Dipietro LV, Wang Y, Chen JJ, Quincy DA (2001). In vitro and in vivo anticancer activities of synthetic macrocyclic ketone analogues of halichondrin B. Cancer Res.

[4] Jordan MA, Kamath K, Manna T, Okouneva T, Miller HP, Davis C, Littlefield BA, Wilson L (2005). The primary antimitotic mechanism of action of the synthetic halichondrin E7389 is suppression of microtubule growth. Mol Cancer Ther.

[5] Cortes J, O'Shaughnessy J, Loesch D, Blum JL, Vahdat LT, Petrakova K, Chollet P, Manikas A, Dieras V, Delozier T, Vladimirov V, Cardoso F, Koh H, Bougnoux P, Dutcus CE, Seegobin S (2011). Eribulin monotherapy versus treatment of physician's choice in patients with metastatic breast cancer (EMBRACE): a phase 3 open-label randomised study. Lancet.

[6] Kaufman PA, Awada A, Twelves C, Yelle L, Perez EA, Velikova G, Olivo MS, He Y, Dutcus CE, Cortes J (2015). Phase III open-label randomized study of eribulin mesylate versus capecitabine in patients with locally advanced or metastatic breast cancer previously treated with an anthracycline and a taxane. J Clin Oncol.

[7] Wilks S, Puhalla S, O'Shaughnessy J, Schwartzberg L, Berrak E, Song J, Cox D, Vahdat L (2014). Phase 2, multicenter, single-arm study of eribulin mesylate with trastuzumab as first-line therapy for locally recurrent or metastatic HER2-positive breast cancer. Clin Breast Cancer.

[8] Komlodi-Pasztor E, Sackett D, Wilkerson J, Fojo T (2011). Mitosis is not a key target of microtubule agents in patient tumors. Nat Rev Clin Oncol.

[9] Mitchison TJ (2012). The proliferation rate paradox in antimitotic chemotherapy. Mol Biol Cell.

[10] Ogden A, Rida PC, Reid MD, Aneja R (2014). Interphase microtubules: chief casualties in the war on cancer?. Drug Discov Today.

[11] Poruchynsky MS, Komlodi-Pasztor E, Trostel S, Wilkerson J, Regairaz M, Pommier Y, Zhang X, Kumar Maity T, Robey R, Burotto M, Sackett D, Guha U, Fojo AT (2015). Microtubule-targeting agents augment the toxicity of DNA-damaging agents by disrupting intracellular trafficking of DNA repair proteins. Proc Natl Acad Sci U S A.

[12] Pourroy B, Honoré S, Pasquier E, Bourgarel-Rey V, Kruczynski A, Briand C, Braguer D (2006). Antiangiogenic concentrations of vinflunine increase the interphase microtubule dynamics and decrease the motility of endothelial cells. Cancer Res.

[13] Honoré S, Pagano A, Gauthier G, Bourgarel-Rey V, Verdier-Pinard P, Civiletti K, Kruczynski A, Braguer D (2008). Antiangiogenic vinflunine affects EB1 localization and microtubule targeting to adhesion sites. Mol Cancer Ther.

[14] Pagano A, Honoré S, Mohan R, Berges R, Akhmanova A, Braguer D (2012). Epothilone B inhibits migration of glioblastoma cells by inducing microtubule catastrophes and affecting EB1 accumulation at microtubule plus ends. Biochem Pharmacol.

[15] Mimori-Kiyosue Y (2011). Shaping microtubules into diverse patterns: molecular connections for setting up both ends. Cytoskeleton (Hoboken).

[16] Marone R, Hess D, Dankort D, Muller WJ, Hynes NE, Badache A (2004). Memo mediates ErbB2-driven cell motility. Nat Cell Biol.

[17] Zaoui K, Honoré S, Isnardon D, Braguer D, Badache A (2008). Memo-RhoA-mDia1 signaling controls microtubules, the actin network, and adhesion site formation in migrating cells. J Cell Biol.

[18] Zaoui K, Benseddik K, Daou P, Salaun D, Badache A (2010). ErbB2 receptor controls microtubule capture by recruiting ACF7 to the plasma membrane of migrating cells. Proc Natl Acad Sci U S A.

[19] Benseddik K, Sen Nkwe N, Daou P, Verdier-Pinard P, Badache A (2013). ErbB2-dependent chemotaxis requires microtubule capture and stabilization coordinated by distinct signaling pathways. PLoS One.

[20] Nakamura S, Grigoriev I, Nogi T, Hamaji T, Cassimeris L, Mimori-Kiyosue Y (2012). Dissecting the nanoscale distributions and functions of microtubule-end-binding proteins EB1 and ch-TOG in interphase HeLa cells. PLoS One.

[21] Maurer SP, Cade NI, Bohner G, Gustafsson N, Boutant E, Surrey T (2014). EB1 accelerates two conformational transitions important for microtubule maturation and dynamics. Curr Biol.

[22] Zanic M, Widlund PO, Hyman AA, Howard J (2013). Synergy between XMAP215 and EB1 increases microtubule growth rates to physiological levels. Nat Cell Biol.

[23] Zhang R, Alushin GM, Brown A, Nogales E (2015). Mechanistic Origin of Microtubule Dynamic Instability and Its Modulation by EB Proteins. Cell.

[24] Smith JA, Wilson L, Azarenko O, Zhu X, Lewis BM, Littlefield BA, Jordan MA (2010). Eribulin binds at microtubule ends to a single site on tubulin to suppress dynamic instability. Biochemistry.

[25] Dabydeen DA, Burnett JC, Bai R, Verdier-Pinard P, Hickford SJ, Pettit GR, Blunt JW, Munro MH, Gussio R, Hamel E (2006). Comparison of the activities of the truncated halichondrin B analog NSC 707389 (E7389) with those of the parent compound and a proposed binding site on tubulin. Mol Pharmacol.

[26] Bai R, Nguyen TL, Burnett JC, Atasoylu O, Munro MH, Pettit GR, Smith AB, Gussio R, Hamel E (2011). Interactions of halichondrin B and eribulin with tubulin. J Chem Inf Model.

[27] Ayaz P, Ye X, Huddleston P, Brautigam CA, Rice LM (2012). A TOG:alphabeta-tubulin complex structure reveals conformation-based mechanisms for a microtubule polymerase. Science.

[28] Prota AE, Bargsten K, Diaz JF, Marsh M, Cuevas C, Liniger M, Neuhaus C, Andreu JM, Altmann KH, Steinmetz MO (2014). A new tubulin-binding site and pharmacophore for microtubule-destabilizing anticancer drugs. Proc Natl Acad Sci U S A.

[29] Lambert JM, Chari RV (2014). Ado-trastuzumab Emtansine (T-DM1): an antibody-drug conjugate (ADC) for HER2-positive breast cancer. J Med Chem.

[30] Watanabe T, Noritake J, Kakeno M, Matsui T, Harada T, Wang S, Itoh N, Sato K, Matsuzawa K, Iwamatsu A, Galjart N, Kaibuchi K (2009). Phosphorylation of CLASP2 by GSK-3beta regulates its interaction with IQGAP1, EB1 and microtubules. J Cell Sci.

[31] Honnappa S, Gouveia SM, Weisbrich A, Damberger FF, Bhavesh NS, Jawhari H, Grigoriev I, van Rijssel FJ, Buey RM, Lawera A, Jelesarov I, Winkler FK, Wuthrich K, Akhmanova A, Steinmetz MO (2009). An EB1-binding motif acts as a microtubule tip localization signal. Cell.

[32] Wen Y, Eng CH, Schmoranzer J, Cabrera-Poch N, Morris EJ, Chen M, Wallar BJ, Alberts AS, Gundersen GG (2004). EB1 and APC bind to mDia to stabilize microtubules downstream of Rho and promote cell migration. Nat Cell Biol.

[33] van der Vaart B, Franker MA, Kuijpers M, Hua S, Bouchet BP, Jiang K, Grigoriev I, Hoogenraad CC, Akhmanova A (2012). Microtubule plus-end tracking proteins SLAIN1/2 and ch-TOG promote axonal development. J Neurosci.

[34] van der Vaart B, Manatschal C, Grigoriev I, Olieric V, Gouveia SM, Bjelic S, Demmers J, Vorobjev I, Hoogenraad CC, Steinmetz MO, Akhmanova A (2011). SLAIN2 links microtubule plus end-tracking proteins and controls microtubule growth in interphase. J Cell Biol.

[35] Kapoor S, Panda D (2012). Kinetic stabilization of microtubule dynamics by indanocine perturbs EB1 localization, induces defects in cell polarity and inhibits migration of MDA-MB-231 cells. Biochem Pharmacol.

[36] Ertych N, Stolz A, Stenzinger A, Weichert W, Kaulfuss S, Burfeind P, Aigner A, Wordeman L, Bastians H (2014). Increased microtubule assembly rates influence chromosomal instability in colorectal cancer cells. Nat Cell Biol.

[37] Vichai V, Kirtikara K (2006). Sulforhodamine B colorimetric assay for cytotoxicity screening. Nat Protoc.

[38] Applegate KT, Besson S, Matov A, Bagonis MH, Jaqaman K, Danuser G (2011). plusTipTracker: Quantitative image analysis software for the measurement of microtubule dynamics. J Struct Biol.

