# Supplementary material for: Eribulin targets a ch-TOG-dependent directed migration of cancer cells
Source: Oncotarget. 2015 Oct 19;6(39):41667–78. doi: 10.18632/oncotarget.6147 (PMC4747180; doi:10.18632/oncotarget.6147)
Supplement: Supplementary file 1 [file oncotarget-06-41667-s001.pdf]

Eribulin Targets a ch-TOG-dependent Directed Migration of Cancer Cells – Chanez et al

Supplemental Materials and Methods

Definition of migration parameters and statistics used in this study

Migration speed: Migration speed is the mean of the sum of all accumulated distances divided by the time of migration (equation 1). The result is given in micrometers per minutes.

Equation 1

$$\text{Migration speed} = \frac{1}{n}\sum_{i=1}^{n}\frac{d_{i,accum}}{\mathrm{t}}$$

Efficiency of forward migration: Potency of each cell to migrate towards the highest concentrations within the gradient of chemoattractant [1]. Tracks were all normalized in a x,y graph with the y axis parallel to the heregulin gradient and oriented positively to the highest concentration of heregulin. We used the y forward migration index (y_{FMI}) which represents the efficiency of the forward migration of cells, in relation to the y-axis (Equation 2).

The larger the index is on an axis, the stronger the chemotactic effect is on this axis. For simplification, it is assumed that the y-axis is parallel to the direction of the chemotactic gradient (Scheme 1).

Equation 2

$$Y_{FMI} = \frac{1}{n}\sum_{i=1}^{n}\frac{Y_{i,end}}{d_{i,accum}}$$

Directness: Ratio between Euclidean distance and accumulated distance (Scheme 1). It represents a measurement of the distance ratio of cell trajectories (Equation 3). Directness (D) is not a direct parameter for assessing chemotaxis but indicates how much cells wander during their migration towards their final position after a fixed duration.

Equation 3

$$\mathrm{D} = \frac{1}{n}\sum_{i=1}^{n}\frac{d_{i,euclid}}{d_{i,accum}}$$

Scheme 1

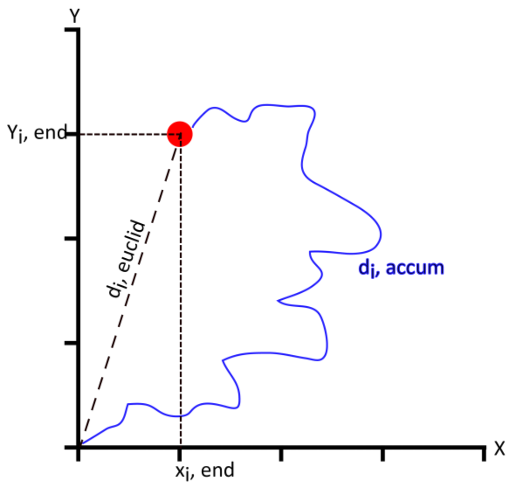

Rose diagram: Circular diagram in which the events (migrated cells in the present case) are represented in sectors with a predetermined angle [2, 3]. The area of each sector is proportional to the number of cell migrating with a final position in this sector.

Rayleigh Test: The Rayleigh test is a statistical test for the uniformity of a circular distribution of points. It depends on the number of object analyzed and their distribution in space. When p-values is smaller than p=0.05, the null hypothesis (uniformity) is rejected and the object have a heterogeneous distribution (due to chemotaxis in the present case) [2, 3].

References

1. Visualization and Data Analysis of Chemotaxis and Chemotaxis and Migration Tool 2.0 in Migration Processes (http://ibidi.com/software/chemotaxis_and_migration_tool/)
2. Statistical Analysis of Circular Data, Fisher NI, Cambridge University Press, 1995, 277 pp
3. Directional Statistics, Mardia KV, Jupp PE, John Wiley & Sons, 2009, 453 pp

Analysis of cell cycle by flow cytometry

SKBr3 cells were grown on collagen for 24h. The medium was replaced by fresh medium containing either DMSO 0.5% alone or eribulin at 0.1, 0.5 or 1 nM and treatment was performed for an additional 4 h or 72 h. At the end of treatment time, culture medium and cells were harvested and pelleted at 800 rpm at 4°C. Each cell pellet was re-suspended in cold 70% ethanol and fixed for 30 min on ice. After centrifugation, fixative was removed and cell pellets were air dried for 15 minutes and re-suspended in PBS. Cells were pelleted and re-suspended in a 1:1 solution of 40 µg/mL propidium iodide (Sigma, St Louis, USA) and 40 µg/mL RNase A (Sigma, St Louis, USA) and incubated 30 min in the dark at 37°C, then kept at 4°C until analysis. The DNA content in each cell nucleus was determined with a LSRFortessa flow cytometer (Becton–Dickinson, San Jose, CA, USA), and the cell cycle was analyzed using FlowJo V10 Software.

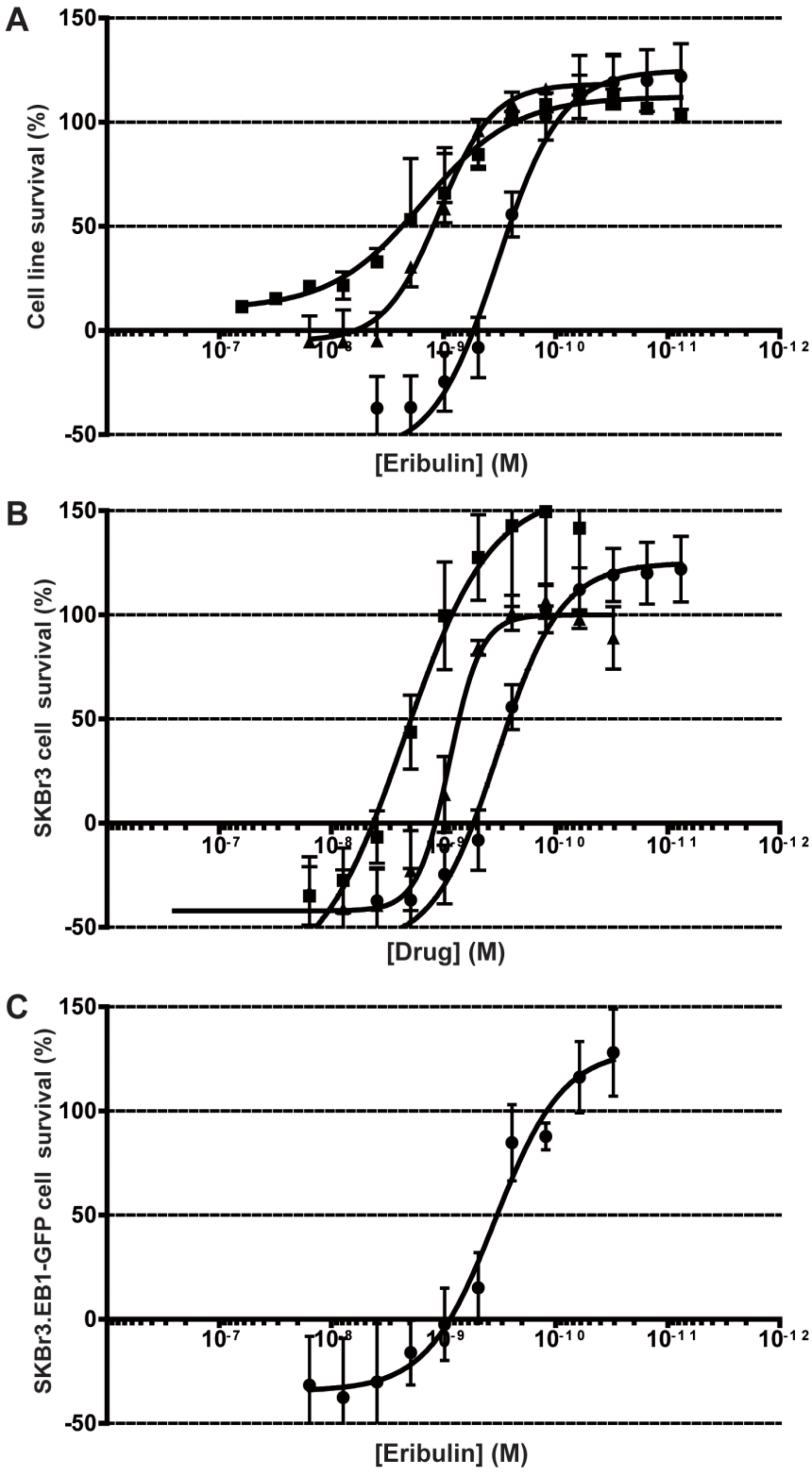

Figure S1. Effect of eribulin on breast cancer cell growth. SKBr3, MDA-MB-231 or T47D cell lines were grown for 72 h in absence or presence of serial dilutions of eribulin, paclitaxel or vinblastine, and their survival was measured using a sulforhodamine B assay. A) Cytotoxicity of eribulin was evaluated in SKBr3 (●), T47D (▲), or MDA-MB-231 (■) cell line. B) Cytotoxicity of eribulin (●), vinblastine (▲) or paclitaxel (■) was evaluated in the SKBr3 cell line. C) Cytotoxicity of eribulin was evaluated in SKBr3.EB1-GFP cells. Percentage of cell relative to control was determined from quadruplicate data points. The average of three independent determinations and SD is presented.

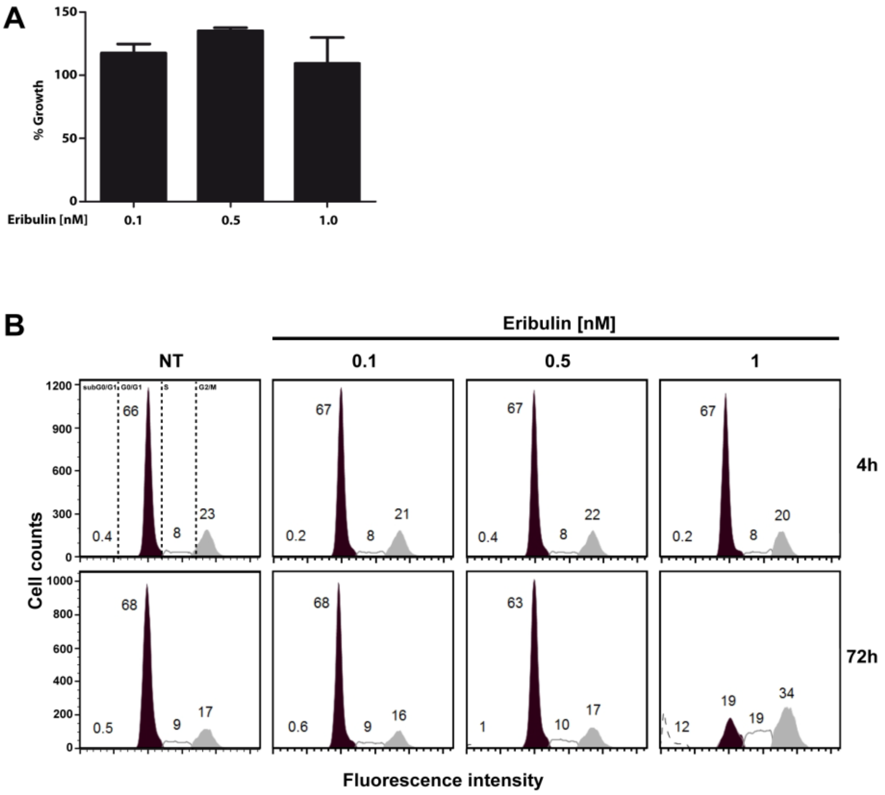

Figure S2. Eribulin treatment of SKBr3 cells for 4 h is not cytotoxic. A) Effect of low concentrations of eribulin after four hours of treatment on SKBr3 cell growth. Cells were treated for 4 h with DMSO only, 0.1, 0.5 or 1.0 nM eribulin and percentage of cell growth determined by a sulforhodamine B assay. The average of three independent determinations and SD is presented. B) Cells were treated for 4 h or 72 h with DMSO only, 0.1, 0.5 or 1.0 nM eribulin and the percentage of cells in sub G0/G1 (dashed line), G0/G1 (black), S (white) and G2/M (grey) phase was determined based on DNA content analyzed by flow cytometry. After 4 h of treatment, concentrations of eribulin up to 1 nM have no impact on cell growth, cell cycle or cell death of SKBr3 cells.

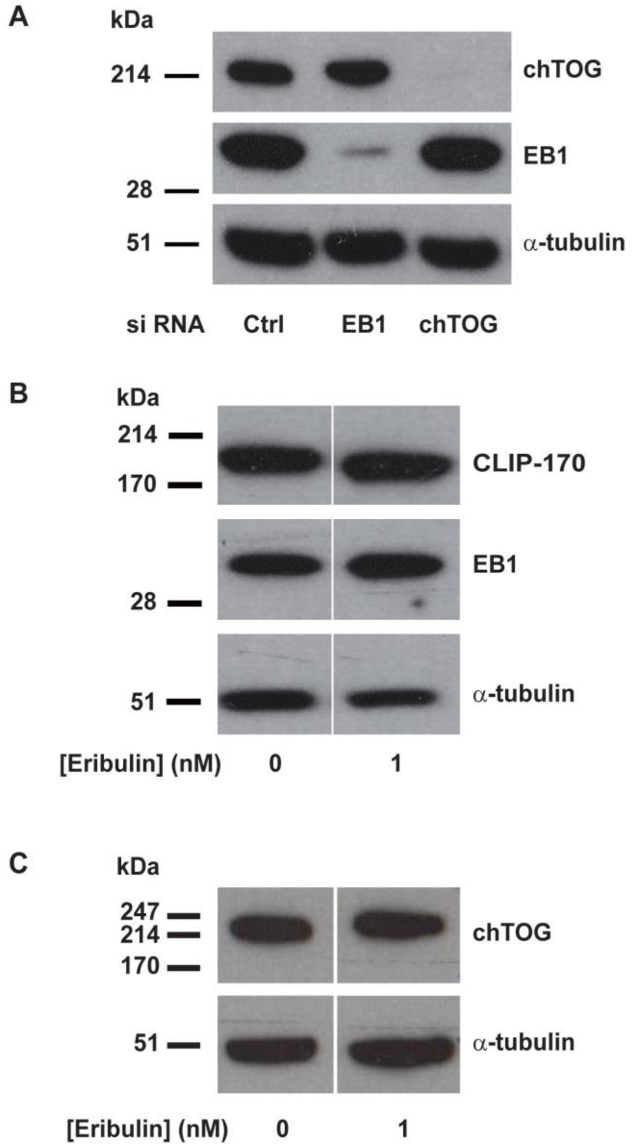

Figure S3. Effect of siRNAs and eribulin treatment on +TIP expression. SKBr3 cells were cultured in the absence or presence of eribulin at 1 nM for 4 h prior to lysis. EB1, CLIP170 and ch-TOG expression was evaluated by Western blotting of cell lysates (30 µg/lane). Tubulin serves as loading control. A) Efficacy and specificity of ch-TOG and EB1 siRNAs at 48h. B-C) Eribulin treatment did not alter the expression levels of EB1, CLIP-170 and ch-TOG.

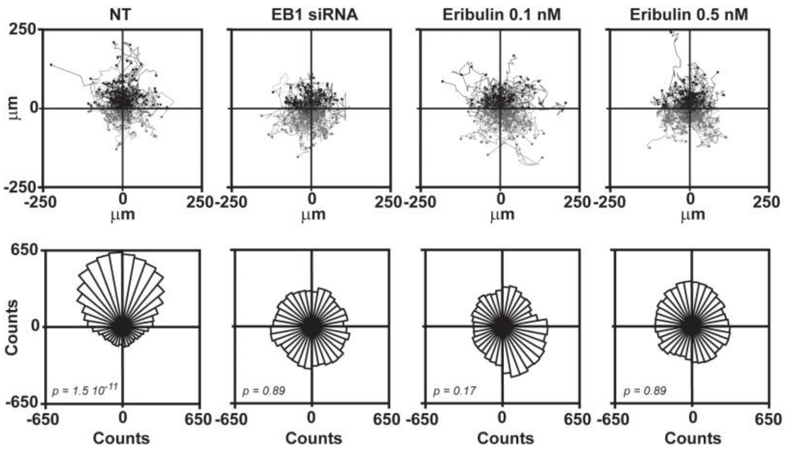

Figure S4. Eribulin inhibits HRG-induced chemotaxis. SKBr3 cells were transfected with a control (Ctrl) or an EB1 siRNA for 48h before tracking of cells in Dunn chambers for 8 h, as they migrated in response to a HRG-gradient (highest concentrations at the top of the figure). Upper panel: tracks of individual cells set to the same origin; cells migrated towards high and low HRG concentrations are represented in black and grey, respectively. Lower panel: rose plots reflecting cell distribution after 8 h of migration; p value < 0.05 indicates unimodal directional cell distribution in the Rayleigh test. NT, non-treated with eribulin. Results from three independent experiments and at least 150 cells are presented.

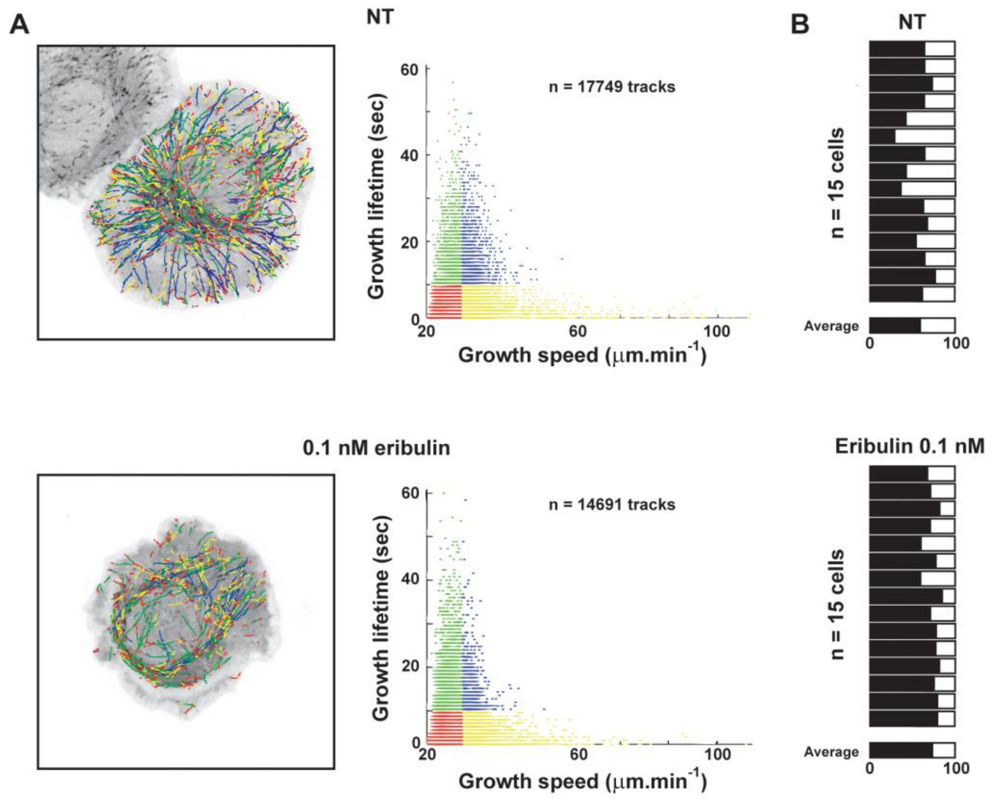

Figure S5. Effects of eribulin on microtubule dynamic parameters. A) EB1-GFP comets were tracked every 60 ms for 1 min in SKBr3 cells pre-treated or not (NT) with 0.1 nM of eribulin for 4 h and resulting tracks were analyzed using +TipTracker. Growth speed (including pauses) and growth lifetime were calculated. 17,749 and 14,691 tracks were analyzed in control and eribulin-treated cell, respectively, in 15 cells from 3 independent experiments. Four categories of tracks are created: slow long lived (green), slow short lived (red), fast long lived (blue) and fast short lived (yellow). Left panels: representative cells with microtubule tracks are shown. Right panels: tracks growth lifetime was plotted relative to microtubule growth speed. B) Because growth lifetime is not changed by 0.1 nM-eribulin treatment (see Figure 4C), tracks were grouped according to growth speed only. Percentage of slow tracks (black) and fast tracks (white) in each cells and in all 15 cells (average) are presented. The percentage of slow growing microtubules is increased upon 0.1 nM eribulin treatment.

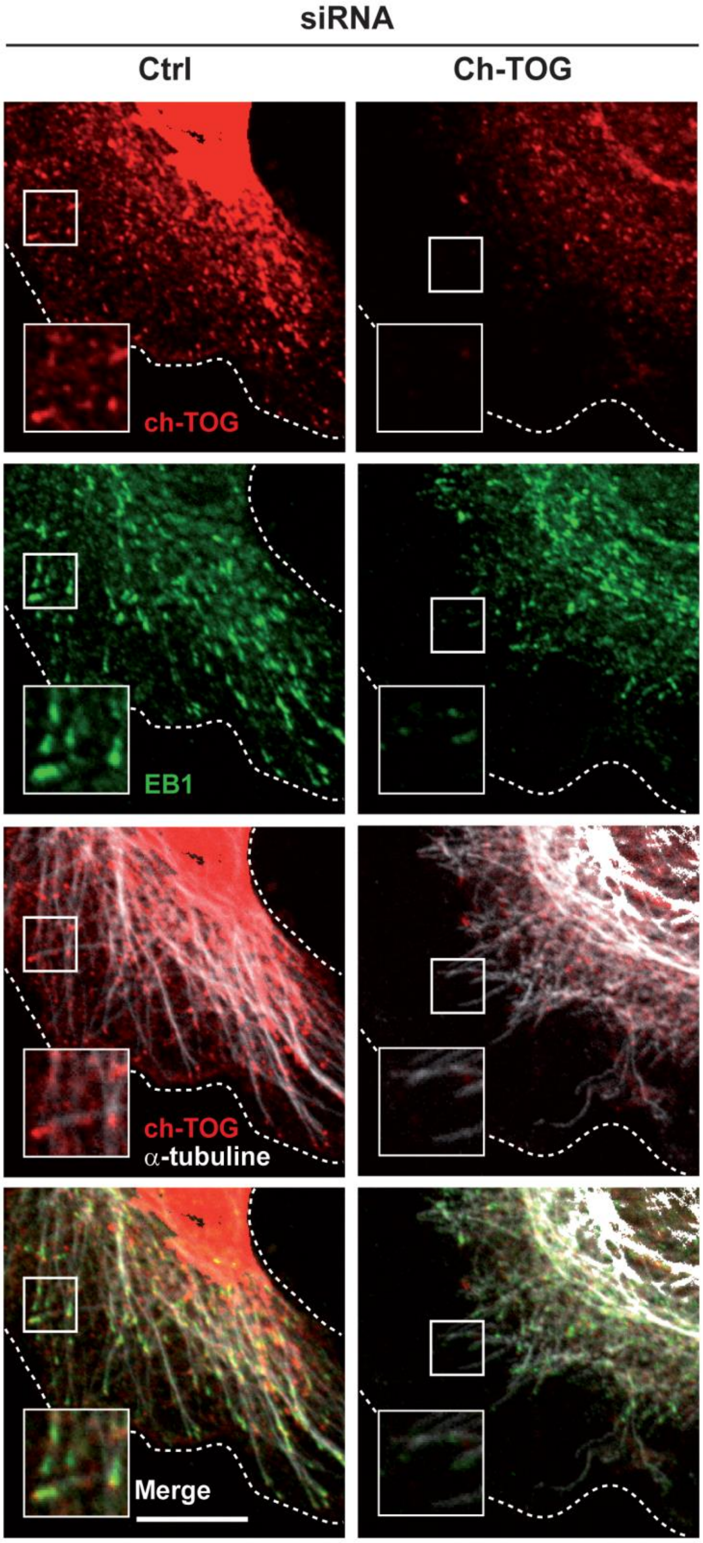

Figure S6. Effect of ch-TOG siRNA on EB1 localization. SKBr3 cells were transfected with a control (Ctrl) or a ch-TOG siRNA for 48 h before addition of HRG for 30 min. EB1, ch-TOG and tubulin localizations were visualized by triple immunofluorescence labeling. White squares show zoomed areas in the cell periphery. Exposure times for the different fluorescence channels were the same for all conditions analyzed. In the presence of ch-TOG siRNA, there is a strong decrease in ch-TOG labeling, but also in the number of EB1 comets relative to control cells. White scale bar represents 10 μm; cell periphery is delineated with dashed lines.

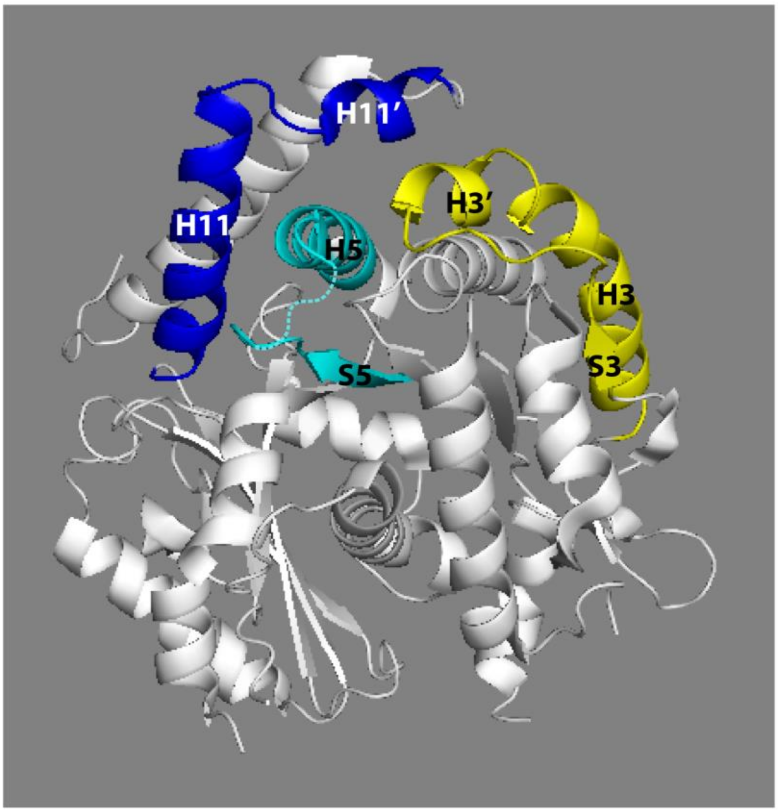

Figure S7. Secondary structures of β-tubulin involved in the binding pockets of eribulin and maytansin. The H11-H11' and H3'-H3 loops involved in eribulin and maytansin binding pockets are represented in dark blue and yellow, respectively; the S5-H5 loop involved in the binding pocket of maytansin is represented in cyan. Note that part of the unresolved S5-H5 loop in PDB 4FFB is materialized by a dashed line with an arbitrary conformation.
